# Supplementary material for: Viral genome sequence datasets display pervasive evidence of strand-specific substitution biases that are best described using non-reversible nucleotide substitution models
Source: eLife. 2025 Sep 30;12:RP87361. doi: 10.7554/eLife.87361 (PMC12483504; doi:10.7554/eLife.87361)
Supplement: Supplementary file 1. [file elife-87361-supp1.docx]

Table S 1 Full details of the datasets used in the study.

| **Genome Type** | **Virus Family** | **Virus Genus** | **Virus Species** | **Dataset Name** |
| --- | --- | --- | --- | --- |
| ssDNA | Circoviridae | Circovirus | Beak and feather disease virus | BFDV |
|  |  |  | Duck circovirus, Goose circovirus | DG_CV |
|  |  |  | Columbine circovirus | PiCV |
|  |  |  | Circovirus | CCCC |
|  |  |  | Bat circovirus | BTC |
|  |  |  | Porcine Circovirus 2 | POCV2 |
|  |  |  | Cyclovirus | CCV |
|  | Geminiviridae | Begomovirus | East Africa cassava mosaic virus, South African cassava mosaic virus | Begomo6 |
|  |  |  | Tomato yellow leaf curl virus | Begomo5 |
|  |  |  | Malvastrum yellow vein Yunnan virus, Cotton leaf curl Multan virus, Bhendi yellow vein India virus | Begomo9 |
|  |  |  | Tobacco yellow dwarf virus, Chickpea chlorosis virus, Chickpea yellows virus | Dicot_1 |
|  |  | Mastrevirus | Chickpea chlorotic dwarf virus | Dicot_2 |
|  |  |  | Maize streak virus | MSV |
|  |  |  | Panicum streak virus | PanSV |
|  |  |  | Wheat dwarf virus | WDV |
|  | Anelloviridae | Annellovirus | Torque teno virus 1 | TTV_1 |
|  |  |  | Torque teno sus virus | TTSV |
|  | Parvoviridae | Aneptorquevirus | minute virus of mice, MVM | MVM |
|  |  | Protoparvovirus | Human parvovirus | HPV |
|  |  |  | Canine parvovirus | CPV |
|  |  |  | porcine parvovirus | PPV |
|  |  | Amdoparvovirus | Carnivore amdoparvovirus | CAV_P |
|  | Nanoviridae | Babuvirus | Banana bunchy top virus | BBTV_M |
|  |  |  |  | BBTV_N |
|  |  |  |  | BBTV_R |
|  |  |  |  | BBTV_S |
|  |  | Nanovirus | Coconut foliar decay virus | CCDV |
|  |  |  | Milk vetch dwarf virus full genome | MDV |
|  |  |  | Pea necrotic yellow dwarf virus | PYDV |
|  |  |  | Faba bean necrotic stunt virus | FBNS |
|  | Microviridae | Microviruses | Microvirus | BMV |
|  | Pleolipoviridae | Pleolipoviruses | Betapleolipovirus | BPV |
|  |  |  | Alphapleolipovirus | APV |
| ssRNA | Astroviridae | Astroviruses | Human astrovirus | HAV |
|  |  |  | Bovine astrovirus | BAV |
|  |  |  | Mamastrovirus | MMV |
|  |  |  | porcine astrovirus | PAV |
|  |  |  | chicken astrovirus | CKV |
|  |  |  | Goose astrovirus | GA |
|  |  |  | Canine astrovirus | CAV_A |
|  | Bromoviridae | Cucumovirus | Cucumber mosaic virus | CMV_RNA1 |
|  |  |  |  | CMV_RNA2 |
|  |  |  |  | CMV_RNA3 |
|  |  | Alphamovirus | Alphalfa mosaic virus | AMS |
|  |  | Cucumovirus | Peanut stunt virus | PSV |
|  | Caliciviridae | Lagovirus | Lagovirus | LAV |
|  |  | coroviruses | Norovirus | NoV |
|  |  | Vesivirus | Vesivirus | VSV |
|  | Closteroviridae | Closterovirus | Citrus tristeza virus | CTV |
|  | Flaviviridae | Flavivirus | Dengue virus | DGV_T1 |
|  |  |  | Japanese encephalitis virus | JEV |
|  | Hepeviridae | Hepevirus | Hepatitis E virus | HPVE1 |
|  |  |  | Hepatitis E2 virus | HPVE2 |
|  | Picornaviridae | Enterovirus | Human Rhinovirus A | HRV_A |
|  |  |  | Enterovirus A | ENV_A |
|  |  | Teschovirus | Techovirus | TCV |
|  |  | Aichivirus | Aichivirus | AiV |
|  |  | Aphthovirus | Foot and mouth disease virus | FMDV |
|  |  |  | Avihepatovirus | AHP |
|  |  | Cardiovirus | Encephalomyo carditis virus | ECV |
|  |  |  | Cardiovirus | CDV |
|  | Fusariviridae | Fusarivirus | Fusariviruses | FRV |
|  | Retroviridae | Lentivirus | Human immuno-deficiency virus 1 | HIV1_setA |
|  |  |  |  | HIV1_M |
|  |  |  |  | HIV1_setC |
|  |  |  |  | HIV1_setD |
|  |  |  |  | HIV1_setE |
|  |  |  |  | HIV1_setF |
|  |  |  | Simian immuno-deficiency virus | SIV |
|  |  |  | Bovine immunodeficiency | BIV |
|  |  |  | Feline immunodeficiency | FIV |
|  |  |  | equine infectious anemia virus | EIV |
|  |  |  | caprine arthritis encephalitis virus | CAV |
|  | Orthomyxo-viridae | Influenzavirus | Influenza virus A | FluA_2 |
|  |  |  | Influenza virus B | FluB_1 |
|  | Filoviridae | Ebolavirus | Ebola virus | Ebola_2 |
|  | Coronaviridae | Merbecovirus | Middle East respiratory syndrome | MERS-COV |
|  |  | Sarbecoviruses | Severe acute respiratory syndrome coronavirus 1 | SARS-COV1 |
|  |  |  | Severe acute respiratory syndrome coronavirus 2 | SARS-COV2 |
|  |  |  | sarbecoviruses | SARB |
| dsDNA | Papillomaviridae | Alphapapillomavirus | Alphapapillomavirus 6 | APPV 6 |
|  |  |  | Alphapapillomavirus 7 | HPV18_2 |
|  |  |  |  | HPV45_2 |
|  |  |  | Alphapapillomavirus 9 | HPV16_2 |
|  |  |  |  | HPV31 |
|  |  |  | Alphapapillomavirus 10 | HPV6_1 |
|  |  |  | Bovine papillomavirus | BPV |
|  |  |  | Lambdaapapillomavirus | LPV |
|  |  |  | Deltapapillomavirus | DPV |
|  |  |  | Xipapillomavirus | XPV |
|  | Polyomaviridae | Polyomavirus | BK polyomavirus | BK_2 |
|  |  |  | JC polyomavirus | JC_2 |
|  |  |  | Bat polyomavirus | BPV |
|  |  |  | Simian virus 40 | SMV_40 |
|  | Caulimoviridae | Caulimovirus | cauliflower mosaic virus | CMV |
|  |  |  | Cacao swollen shoot virus | CSSV |
|  |  |  | Strawberry vein banding virus | SVBV |
|  |  |  | Dioscorea bacilliform AL virus | DBAV |
|  |  |  | Rice tungro bacilliform virus | RTBV |
|  |  |  | badnavirus | BDV |
|  | Siphoviridae | Escherichia virus Lambda | coliphage lambda | CLV |
|  | [Tectiviridae](http://www.virology.net/big_virology/BVDNAtecti.html) | Tectivirus | Tectivirus | TTIV |
|  | [Adenoviridae](http://www.virology.net/big_virology/BVDNAadeno.html) | Aviadenovirus | Fowl aviadenovirus C | FAV_C |
|  |  |  | Fowl aviadenovirus E | FAV_E |
|  |  |  | Fowl aviadenovirus A | FAV_A |
|  |  |  | Fowl aviadenovirus D | FAV_D |
|  |  | Mastadenovirus | Human mastadenovirus B | HMAV_B |
|  |  |  | Human mastadenovirus D | HMAV_D |
|  |  |  | Human mastadenovirus C | HMAV_C |
|  |  |  | Human mastadenovirus E | HMAV_E |
| DsRNA | Birnaviridae | Avibirnavirus | Gumburo virus_setA | GBV_A |
|  |  |  | Gumburo virus_setB | GBV_B |
|  |  | Aquabirnavirus | Infectious pancreatic necrosis virus | IPNV |
|  |  |  | Aquabirnavirus | AQBV |
|  | Reoviridae | Orbivirus | Bluetongue_virus_setA | BTV_A |
|  |  |  | Bluetongue_virus_setB | BTV_B |
|  |  |  | Bluetongue_virus_setC | BTV_C |
|  |  |  | Bluetongue_virus_setD | BTV_D |
|  |  |  | Bluetongue_virus_setF | BTV_F |
|  |  |  | Bluetongue_virus_setG | BTV_G |
|  |  |  | Bluetongue_virus_setH | BTV_H |
|  |  |  | Bluetongue_virus_setI | BTV_I |
|  |  | Rotavirus | Bovine_rotavirus_A_setC | BRVA_C |
|  |  |  | Human_rotavirus_A_setA | HRVA_A |
|  |  |  | Human_rotavirus_A_setB | HRVA_B |
|  |  |  | Human_rotavirus_A_setC | HRVA_C |
|  |  |  | Human_rotavirus_A_setD2 | HRVA_D2 |
|  |  |  | Human_rotavirus_A_setE | HRVA_E |
|  |  |  | Human_rotavirus_A_setF | BRVA_F |
|  |  |  | Human_rotavirus_A_setG | HRVA_G |
|  |  |  | Human_rotavirus_A_setH | HRVA_H |
|  |  |  | Porcine_rotavirus_A_setA | PRVA_A |
|  |  |  | Porcine_rotavirus_A_setB | PRVA_B |
|  |  |  | Human_rotavirus_C_setA | HRVC_A |
|  |  | Orthoreovirus | Pteropine orthoreovirus | PTOV |
|  |  | Fijivirus | Fijivirus_setB | FJV_B |
|  | Totiviridae | Totivirus | Totivirus | TTV |
|  |  | Giardiavirus | Giardiavirus | GDV |
|  | Hypoviridae | Hypovirus | Hypovirus | HPV |
|  | Endornaviridae | Endornavirus | Endornavirus | EDV |
|  |  | Alphaendornavirus | Bell pepper alphaendornavirus | BPAV |
